# Preventive effect of cyproheptadine hydrochloride in refractory patients with frequent migraine

**DOI:** 10.1186/2193-1801-2-573

**Published:** 2013-10-29

**Authors:** Hirohisa Okuma, Kazuyuki Iijima, Takashi Yasuda, Kentaro Tokuoka, Yasuhisa Kitagawa

**Affiliations:** Department of Neurology, Tokai University Hachioji Hospital, 1838 Ishikawa-cho, Hachioji city, Tokyo 192-0032 Japan

## Abstract

Cyproheptadine hydrochloride (CH) is rarely used to treat adult patients with migraine in Japan because it causes sleepiness. In this study, we investigated the preventive effect of CH in 12 patients who had failed to respond to conventional preventive treatments among 103 migraine patients treated at our hospital. These 12 subjects had all received unsuccessful migraine prophylaxis with lomerizine, valproic acid and topiramate, or had discontinued these treatments due to adverse reactions. Initially, the subjects were given 4 mg CH before sleeping. In those who experienced no clinically significant sleepiness following the treatment, the drug was orally administered at 4 mg after breakfast as well (8 mg per day in total). Drug efficacy was evaluated by examining the frequency of migraine at one month and three months after the start of treatment. The frequency of migraine was dramatically reduced in all patients within 7 to 10 days after starting treatment. The average frequency of migraine during the three-month period was 2.6 episodes per month, representing a significant (p < 0.01) reduction from the pretreatment frequency of over 10 per month. Our results indicate that CH may be effective as a migraine-preventive treatment for patients in whom conventional drugs have been ineffective or have caused side effects. But this study is not a double blind randomized trial, and an open study with no control group.

## Introduction

In patients who experience frequent migraine attacks, the risk of rebound headache is quite high, and regardless of the efficacy of the rescue medication, there is a need for preventive therapy (Headache Classification Committee of the International Headache Society 2004). Cyproheptadine hydrochloride (CH) is generally used outside Japan as a migraine preventive treatment in pediatric patients (Kara 2010), with excellent results. In Japan, antidepressants, beta-blockers, antiepileptic agents, calcium antagonists, etc., are the main medications used to prevent migraine in adults (Pascual 2012; Tfelt-hansen 2013), and CH is rarely used in adults, because it causes sleepiness. Consequently, there has been no report on the effectiveness of this drug in adult patients with migraine in Japan. Although CH as an antihistamine has been largely replaced by nonsedating compounds, it nevertheless has efficacy in preventing migraine attacks (Rao et al. 2000; Lewis et al. 2004), and not just in patients whose migraines are worsened or triggered by underlying allergies. In this study, we investigated the preventive effect of CH in patients who did not respond to conventional preventive treatments.

### Subjects

The migraine preventive effect of CH was investigated in 12 of 103 migraine patients treated at our hospital. These 12 subjects (1 men and 11 women, average age 35; Table 1) had all received unsuccessful migraine prophylaxis with lomerizine, valproic acid and topiramate, or had discontinued such treatments due to adverse reactions or because of possible fetal side effects during pregnancy. The frequency of their migraine attacks was 10 or more per month. Among these patients, four had migraine with aura, four had migraine without aura, and four had menstrual migraine.

**Table 1:** **Results of the use of cyproheptadine hydrochroride for in refractory migraine patients prophylaxis**

n = 12	Dose of cyproheptadine hydrocride	
	4 mg/day (n = 8)	8 mg/day (n = 4)
Gender	male: female 1:7	all female
Average age	34 ± 6	36 ± 5
Frequency of migraine premedication	8.7/month	10.6/month
Frequency of migraine one month after start of treament	1.6/month	1.2/month
Frequency of migraine three month after start of treatment	3.1/month	2.1/month
Adverse reactions	Body weight 28.6%	Body weight 25.0%
	Sleepiness 14.3%	Sleepiness 50.0%

## Methods

CH was orally administered in subjects who had received unsuccessful migraine prophylaxis with lomerizine hydrochloride, valproic acid, and topiramate, or who had encountered side effects with such treatments, or had contraindications. Because CH may cause sleepiness, CH 4 mg was initially given before sleeping. In those who experienced no clinically significant sleepiness following the treatment, the drug was orally administered at 4 mg after breakfast as well. Drug efficacy was evaluated by examining the frequency of migraine at one month and three months after the start of treatment. But this study is not a double blind randomized trial, and an open study with no control group.

## Results

**Figure 1:**
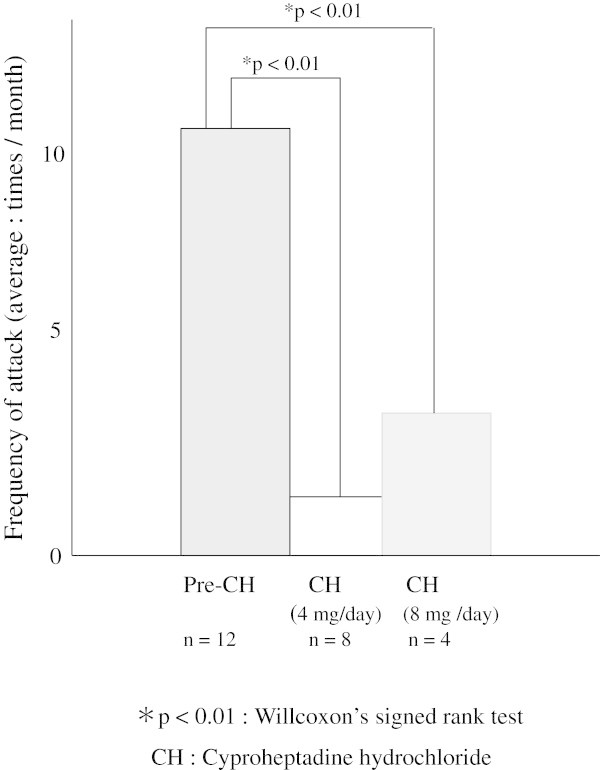
**The results of the Willcoxon’s signed rank test of the use of cyproheptadine hydrochroride.**

The frequency of migraine was dramatically reduced in all patients within 7 to 10 days after starting treatment. No migraine attacks were observed in 9 of the 12 patients during 1 month after starting the drug. In two other patients, the frequency of monthly migraine attacks was reduced to 1. The average frequency of migraine attacks during three months was 2.6 times per month. These results indicate that CH is effective for preventing migraine. The incidences of adverse reactions were 60.7% for sleepiness and 30.3% for increased appetite. Sleepiness was alleviated by changing the dose regimen to administration of 1 tablet (4 mg) before sleeping. Weight gain caused by increased appetite did not develop into a problem after subjects were advised to pay more attention to their diet. The average frequency of migraine attacks before CH administration was 8.7 times per month, but this was decreased to 3.1 times per month at 3 months after the start of treatment in the group that received 4 mg per day. The average frequency of migraine attacks before CH administration was 10.6 times per month in the group that received 8 mg per day, but this was decreased to 2.1 times per month after 3 months. Weight gain was similar in both groups, but sleepiness was more marked in the higher dose group, in which its incidence reached 50% (Table 1). The results of the Willcoxon’s signed rank test were as follows. Two groups had a ρ-value of 0.01, and showed a significantly greater contribution than the premedication (Figure 1).

## Discussion

A double-blind study for the migraine prophylaxis by propranol and CH is reported. Before (Rao et al. 2000), but our study, we performed all prophylaxis to the last, but protective efficacy for the migraine uses CH only to refractory patients with frequent migraine. Therefore, it was shown to our study that CH was effective for the refractory patients with frequent migraine.

Involvement of the trigeminal-vascular system (Mathew 2001; Silberstein 2004) is generally considered the most likely cause of migraine. According to this theory, release of serotonin from platelets induces cerebral vasoconstriction and decreases cerebral blood flow, inducing aura, including scintillating scotomata. Subsequent serotonin depletion leads to cerebral vasodilatation and stimulation of the trigeminal nerve, which extends around vessels such as intracranial large vessels and the dura mater, inducing neurogenic inflammation and thus triggering migraine (Ollat 1992; Greek 2006; Hammon & Hoyer 2008). There are seven types of serotonin receptors. The serotonin 1 and 2 receptors are involved in cerebral vasoconstriction, and the serotonin 1 receptor exists predominantly in cerebral blood vessels. Furthermore, several subtypes of the serotonin 1 receptor have been identified, and among them, the serotonin 1B and 1D receptors are considered to be involved in cerebral vasoconstriction and in causing migraine (Ferrari et al. 2001; Wolff et al. 2003; Filip & Bader 2009). It has also been shown that binding of serotonin to the serotonin 1D receptor at trigeminal nerve terminals inhibits release of vasoactive peptides, including calcitonin gene-related peptides.

CH, which we used in this study, is generally used for migraine in pediatric patients, but not adult patients. In addition to its antihistaminic action, CH exerts antiserotonin and anticholinergic actions, and antagonizes histamine and serotonin receptors. It does not inhibit or chemically inactivate release of histamine and serotonin, but competitively and reversibly antagonizes histamine and serotonin at receptor sites. Therefore, we conclude that CH showed superior prophylactic efficacy to commonly used drugs in our patients owing to its inhibition of the release of vasoactive peptides, including calcitonin gene-related peptides, with simultaneous inhibition of both serotonin 1B and 1D receptors and prevention of neurogenic inflammation caused by stimulation of the trigeminal nerve in patients with refractory migraine (Villalon & Olsen 2009). Its greater efficacy in patients with allergic rhinitis and refractory migraine is considered to be attributable its antihistaminic action.

Based on the present results, we suggest that even adult patients who do not respond to multiple drugs currently used for migraine prophylaxis should be given preventive therapy with CH. However, since the antiserotonin action of CH is stronger than that of lysergic acid diethylamide (LSD), great care is needed to prevent the onset of visual hallucinations and confusion, especially at higher doses. Also, weight gain should be anticipated due to stimulation of the feeding center by the anticholinergic action of this drug. Long-term treatment with CH should be avoided.

## Conclusions

Currently, antidepressants, beta-blockers, antiepileptic agents, and calcium antagonists are used in Japan to prevent frequent, intractable migraine in adults. The results of this study demonstrate that CH is effective as a migraine-preventive treatment for adult Japanese patients in whom such conventional drugs are ineffective or induce undesirable side effects.
